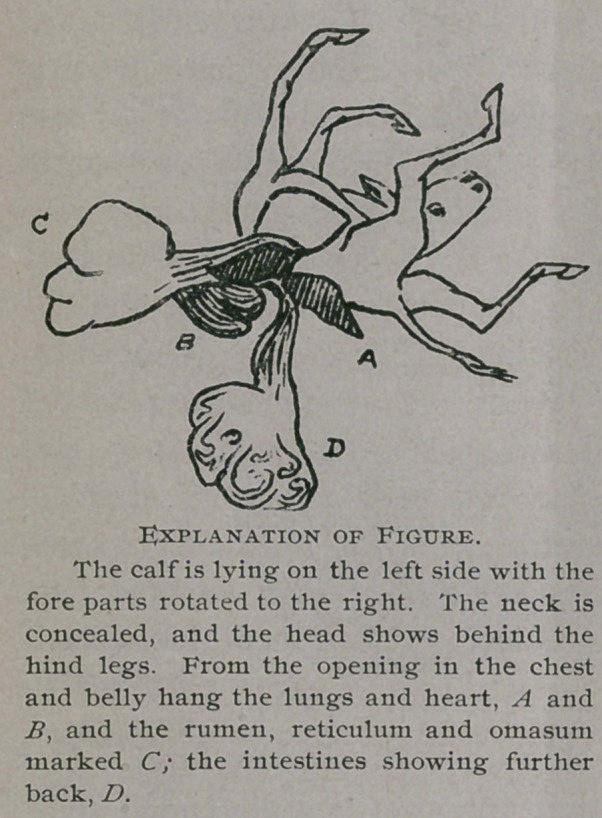# A Peculiar Monstrosity

**Published:** 1891-01

**Authors:** Daniel D. Lee

**Affiliations:** Instructor in Anatomy, Veterinary Department, Harvard University


					﻿A peculiar monstrosity.
By Daniei, D. Lee, M.D.V.,
Instructor in Anatomy, Veterinary Department, Harvard
University.
I was called to see a valuable Jersey cow that had just calved,
and found all the men on the place, in the absence of the owner,
very much excited.
The calf was what is known as a celosomian monster, that is,
one having a ruptured body.
I found on weighing the creature that it reached 47 lbs.; it
was well developed and proportioned, and had its first teeth
through the gums.
The spinal column was bent downward in the centre and
upward at each extremity, thus
forming a deep depression on
the upper surface of the body.
The thorax and abdomen
had never closed, and in place
of the parietes there was a large
diamond-shaped opening.
To the skin at the edge of
this opening were attached the
foetal envelopes, and from it pro-
truded all the thoracic and ab-
dominal viscera, all of which,
as well as the head and limbs,
were perfectly developed, al-
though bent and twisted in all
manner of positions, as may be
seen by the photograph. The
sex was female.
Fleming says that such cases are quite frequent, especially
in cows, and cites a number; in none of them do the creatures
seem to be as complete in all their parts as in this case.
The cow required no assistance, and never licked the crea-
ture or paid any attention to it; she was supposed by the men on
the place to have attacked the calf and maimed it, and they, sup-
posing her to be mad, had sent for me.
				

## Figures and Tables

**Figure f1:**